# Laryngotomy Successfully Performed in a Case of Foreign Body in the Larynx

**Published:** 1852-04

**Authors:** G. R. Morehouse

**Affiliations:** Philadelphia


### *Laryngotomy successfully performed in a case of Foreign Body in the Larynx.* By G. R. Morehouse, M. D., Philadelphia.

To the Editors of the Medical Examiner.

*Gentlemen,*—I send you the history of the case of "Foreign Body in the Larynx," upon which I recently operated.

The little patient, a girl of ten years, daughter of Mrs. Saunders, on Wednesday morning, February 26th, while laughing and romping with her school-fellows, drew into the larynx a piece of almond-shell, which she had been holding in her mouth. She was immediately seized with a prolonged paroxysm of coughing, followed by dyspnœa and loss of voice. As soon as aid could be obtained, she was carried home, and Drs. Scoffin and Gegan successively sent for. Under their hands every medical means for disengaging the shell was tried; the œsophagus also was explored, and instruments used to force any thing contained therein into the stomach; but every effort seemed in vain. The dyspnœa increased and the paroxysms of cough became more incessant and more fatiguing. The only remaining mode of relief was the removal of the foreign body, through an opening in the windpipe. Accordingly, on Thursday afternoon, I was called to see the child and operate, if advisable. At that time the condition of the little sufferer proclaimed the necessity for immediate aid. The face was turgid and of a purplish hue, the eyes protruding, and slightly divergent, the mouth circled with a white zone, and the nostrils distended and pale, forming a countenance expressive of mingled anxiety and despair, the head was inclined forward, and the muscles of forced inspiration strongly contracted, giving to the neck the appearance of great emaciation. Deglutition seemed unimpaired, and she was able, with apparent ease, to gratify her craving thirst. Respiration was with difficulty maintained, and was accompanied with a wheezing sound, similar to that heard in asthma, although much more feeble. She complained of no pain, except when lateral pressure was made on the larynx. She seemed disinclined to exertion, except at intervals, when her motions were hurried and restless. She seemed fully to appreciate what was passing around her, and spoke to her friends in whispers, repeatedly expressing a wish to die.

Upon auscultation, the respiratory murmur, although barely discernible, was found alike free in either lung; there was no rattling sound to indicate the presence of a loose body in the trachea. Over the larynx, however, a whistling sound, as of a person blowing through a quill, was distinctly heard. These facts, therefore, the incessant cough, the dyspnœa, the pain on pressure, the whispering voice, and the whistling sound of constriction, all pointed to the ventricles of the larynx as the position occupied by the foreign body. By the time that these necessary examinations were made, and the mother's consent to the operation obtained, it had become too dark to operate without the aid of artificial light. The extreme condition of the patient, however, rendered it necessary that an opening, at least, should be made in the windpipe, to prevent death from apnœa. The operation of laryngo-tracheotomy, as originally suggested by Boyer, was selected as most appropriate. The patient was placed upon the table, and an attempt made to commence the operation. The violent convulsive struggles of the child, and the forced forward flexion of the head, rendered it impossible to proceed. Ether was therefore administered, sufficient to quiet, without producing its full anæsthetic effect. The superficial tissues were then divided in the middle line, by a cut nearly three inches in length, the isthmus of the thyroid body was separated from its attachment, and the plexus of thyroid veins pushed aside with the handle of the scalpel. The windpipe thus being laid bare, and the hæmorrhage slight, as soon as the effect of the ether had passed away, the knife was entered just below the crico-thyroidean artery, dividing, as it was withdrawn, the cricoid cartilage and three upper rings of the trachea. The air rushed from the wound as soon as the knife was removed, blowing before it the blood which flowed from the cut. The patient was raised and placed in a position favorable for the outward flow of the blood.

The relief experienced by the child was immediate and most gratifying, the black blood sunk from the face, the asthmatic breathing ceased, the laboring muscles were at rest, and the countenance, a moment since livid, swollen and inexpressive, was now lit up with a smile of gratitude. As soon as oozing had ceased, examination was made for the foreign body, and it was found effusion had taken place in the sub-mucous tissue, almost obliterating the cavity of the trachea at its upper portion. So great was the swelling that it was impossible to introduce an instrument through the rima glottidis, with the hope of extricating the shell; it was, therefore, proposed to continue the incision upward and divide the thyroid cartilage. On account of the prostrate condition of the child, however, and in hope that the œdema would subside and render the extension of the operation unnecessary, it was concluded to defer the division of the cartilage until the morning. In the mean time a conical curved tube, flattened laterally, was introduced through the opening, and secured by means of tapes passed round the neck. An anodyne was administered, and the patient left for the night.

On the following morning, the appearance of the child was propitious, she had rested well during the night, being troubled but little with cough; the tube had occasionally clogged with mucus, but was readily cleared with a feather. The œdema in the trachea had greatly subsided; the chords were, however, in apparently as close proximity as ever. It was decided, therefore, to open the thyroid cartilage, and for this purpose a probe-pointed bistoury was prepared in the following manner. A coating of wax was applied to the sides of the blade, thus converting it into an edgeless instrument, which having passed between the lips of the *rima*, would glide along without injuring them, to their junction with the thyroid cartilage. The bistoury thus prepared was entered at the previous opening, passed between the chords, and with it the cartilage divided. The crico-thyroidean artery was cut, but was readily sealed by the application of a point of caustic. The cartilage was pressed asunder and the piece of shell removed from the left ventricle, by means of a pair of polypus forceps. The tube was permitted to remain in the wound until the swelling of the glottis had subsided, which could readily be judged of by the facility of breathing when the tube was closed. On the second day the tube was removed, and the wound closed with stitches and adhesive plaster. In order that the sides of the wound might be brought in apposition throughout their whole depth, a compress of a double headed roller was employed, the roller (half-an-inch in diameter) pressing parallel to the cut on either side, and bound to the neck by means of a bandage.

The cut has healed kindly and rapidly; the water dressing was used, and parts of the surface occasionally touched with nitrate of silver. The wound was entirely healed, on March 12th, fifteen days after the operation, the child's voice is as clear as ever in the morning, but towards evening is somewhat husky, especially if she is disobedient and talks much during the day.

